# Unusual modes of cell and nuclear divisions characterise *Drosophila* development

**DOI:** 10.1042/BST20231341

**Published:** 2024-11-07

**Authors:** Qiaolin Yang, Fernando Wijaya, Ridam Kapoor, Harshaa Chandrasekaran, Siddhant Jagtiani, Izaac Moran, Gary R. Hime

**Affiliations:** Department of Anatomy and Physiology, University of Melbourne, Melbourne, VIC 3010, Australia

**Keywords:** cell proliferation, cellular reproduction, cytokinesis, developmental biology, DNA replication and recombination, *Drosophila melanogaster*

## Abstract

The growth and development of metazoan organisms is dependent upon a co-ordinated programme of cellular proliferation and differentiation, from the initial formation of the zygote through to maintenance of mature organs in adult organisms. Early studies of proliferation of *ex vivo* cultures and unicellular eukaryotes described a cyclic nature of cell division characterised by periods of DNA synthesis (S-phase) and segregation of newly synthesized chromosomes (M-phase) interspersed by seeming inactivity, the gap phases, G1 and G2. We now know that G1 and G2 play critical roles in regulating the cell cycle, including monitoring of favourable environmental conditions to facilitate cell division, and ensuring genomic integrity prior to DNA replication and nuclear division. M-phase is usually followed by the physical separation of nascent daughters, termed cytokinesis. These phases where G1 leads to S phase, followed by G2 prior to M phase and the subsequent cytokinesis to produce two daughters, both identical in genomic composition and cellular morphology are what might be termed an archetypal cell division. Studies of development of many different organs in different species have demonstrated that this stereotypical cell cycle is often subverted to produce specific developmental outcomes, and examples from over 100 years of analysis of the development of *Drosophila melanogaster* have uncovered many different modes of cell division within this one species.

Although this review will focus on unusual modes of cell division in adult *Drosophila* tissues, including their maintenance within the adult organism and their development from immediate larval and pupal precursors, some discussion is warranted of embryonic and larval cell division, as different division types are also observed during these developmental periods.

## The early *Drosophila* embryo

**Figure 1.**
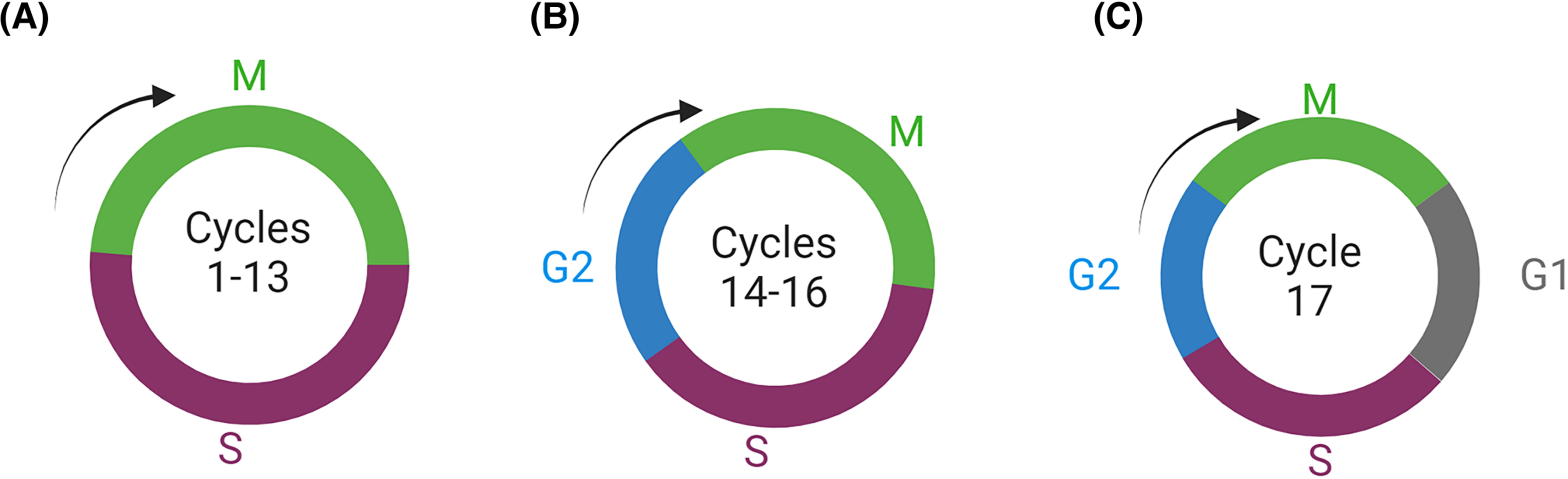
The early embryo — cell cycles. Cell cycle modes and lengths vary during embryonic development. All cycles up to cycle 14 occur in a syncytium and are purely nuclear cycles consisting of alternating S and M phases. After formation of the cellular blastoderm during cycle 14 a G2 phase is acquired which is maintained until cycle 17 when a more typical cell cycle is initiated with the addition of a G1 phase.

The combining of haploid male and female genomes, or syngamy, is frequently described as a fusion of male and female pronuclei but organisms differ in the way that diploidy is achieved. *Drosophila* syngamy is gonomeric, which refers to the male and female genomic contributions remaining in separate groups while aligning to the first mitotic spindle and only intermingling during telophase [1], reviewed in [2]. As in many species, the next series of cleavage divisions are rapid and synchronous. The first 13 nuclear divisions have the shortest cycles known in eukaryotes, ranging from 8.3 to 23 min, with longer cycles observed as the cleavage stage progresses [3,4]. These rapid divisions provide the undifferentiated tissue from which the embryo is patterned and also allow embryonic development to be completed within a 24-h period. This may be important for an egg that is laid on the surface of a food source as it shortens the time until the mobile larva can burrow into the food and facilitate some protection from predation. The speed of cleavage divisions is due to a combination of (a) deposition of maternal factors into the oocyte, such that transcription is not necessary until the mid-blastula transition [5], (b) extremely rapid DNA replication [6,7], and (c) the essentially unregulated cell cycles that are comprised of alternating S- and M-phases (Figure 1), without G1 or G2 [8]. The other unusual characteristic of these early divisions is that they occur within a syncytium [3], with a cell membrane surrounding the large yolk-filled embryo and the cleavage divisions proceeding as nuclear division cycles. It is only after cycle 13, when all of the nuclei have migrated to the periphery of the embryo that cell membranes grow down between each nucleus to generate the individual cells that comprise the cellular blastoderm [9]. The next cell cycle, the first cellular mitosis, is an asynchronous cycle comprising G2, S and M-phases (Figure 1), and the patterns of division correspond to generation of organ primordia within the embryo [9]. Thus, regulation of mitosis can be considered one of the first initiators of organogenesis. The timing of these asynchronous divisions is directly related to regulation and accumulation of the String protein, a Cdc25 phosphatase orthologue that increases Cdk (cyclin-dependent kinase) activity within the Cdk-cyclin complex by dephosphorylation of an inhibitory site within the M cyclin and drives the cell into M phase [10,11]. The differing modes of nuclear and cell division in the embryo therefore permit rapid development prior to morphological differentiation, and a means of segregating cells into discrete developmental units. The developmental timing of these modes could be considered a function of the basic units of morphology (nuclei vs. cells), the genetic regulation of these units of morphology (maternal vs. zygotic) and availability of biochemical regulators (e.g. dilution of maternal products as cleavage proceeds).

## Larval growth and polyploidy

During larval development mitosis facilitates an increase in size of the central nervous system and imaginal discs but many tissues undergo variant cell cycles and become polyploid. These tissues generally cannot divide further by mitosis but induction of polyploidy can result in an increase in the size of individual cells [12–15]. As most larval tissues are histolysed during metamorphosis [16] they have the capacity to become terminally post-mitotic and not interfere with further development of the adult organism [17]. Initiation of tissue histolysis during metamorphosis may be associated with elevated autophagy being correlated with a higher degree of polyploidy [18]. It would be interesting to compare levels of polyploidy in hemimetabolous and holometabolous (which undergo a pupal moult) insects to determine if the developmental strategy of metamorphosis, which results in many larval tissues that are not carried into the adult body plan, is associated with general higher rates of polyploid tissues.

**Figure 2.**
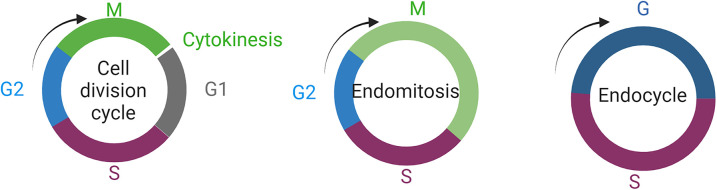
Larval growth and polyploidy — endoreplication. The archetypal mitotic cell cycle division cycle completes separation of daughter cells via cytokinesis following mitosis, and generates daughter cells with identical genomic content to the parent cell. Polyploidy can result from either endomitosis, whereby cells undergo an abortive mitosis that does not feature cytokinesis, or via an endocycle consisting of multiple S phases alternating with a gap phase without entering mitosis. Image was generated with Biorender.com.

The most well-known example of polyploidy in development are the larval salivary glands. Cells of the salivary glands contain the famous polytene chromosomes that were instrumental for early gene mapping studies [19–24]. Salivary gland cells increase DNA content via endoreplication — a phenomenon where DNA replication occurs without either cytokinesis or nuclear division, hence increasing the number of copy of chromosomes [25]. There are two proposed mechanisms in which this abnormal cell cycle is achievable: endomitosis and endocycling [26]. Cells undergoing endomitosis proceed through G1, S, G2 and M phases normally, but skip cytokinesis (Figure 2) to produce multinucleate cells [27], or may alter M phase to proceed through anaphase A but not anaphase B to produce cells that each contain a single polyploid nucleus [15,28]. Endocycling, such as observed in salivary gland cells, involves cells skipping M phase entirely (Figure 2) and cycling between the G and S phases [29–31].

Endoreplication has been linked to alternate fluctuations of Cdks and cyclins during the cell cycle [32,33]. The association of mitotic cyclins with Cdk1 promotes entry to mitosis while activation of Cdk2 by S-phase cyclins drive entry into S phase [34]. In *Drosophila*, Cyclin E acts as the primary S-phase cyclin that binds to Cdk2 [35]. In endocycling cells, there is only a very low level of mitotic cyclins that also have other roles in cell growth, and oscillating levels of Cyclin E [30,36] that promote new rounds of DNA replication.

One hypothesis as to why endoreplication and subsequent polyploidy is observed in many larval tissues is that it permits tissues growth while maintaining cellular barriers. For example, the larval central nervous system maintains a blood brain barrier (BBB) with circulating hemolymph via specialised glial cells known as subperineurial glia (SPGs) [37]. The larval brain increases in size during larval development due to a pool of neural stem cells that produce both nascent neurons and glia. This increase in tissue volume requires that the SPGs also increase the area that they must cover to maintain the BBB but if they were to also proliferate via mitosis followed by cytokinesis there would entail temporary breaks to the BBB. Instead, the SPGs undergo endoreplication and thereby increase in cell size, that can cover more area, and maintain the septate junctions that facilitate the BBB [15,38]. This also illustrates the nature of a generally observed conservation of nucleus : cytoplasm ratio. As the nuclear volume increases due to an increase in genomic mass there is a compensatory increase in cytoplasmic volume [39,40].

## Polyploidy in adult tissues

Several adult tissues contain polyploid cells apart from the SPGs that continue to maintain the BBB in post-eclosion flies [15,26,38,41–50]. Main body follicle cells which encapsulate growing egg chambers, composed of an oocyte paused in mid-meiosis connected via ring canals to fifteen nurse cells, undergo three rounds of endoreplication [51] but their accompanying nurse cells complete multiple rounds of endoreplication, resulting in a ploidy of 1500C [50] comparable to larval salivary gland cells [52]. The nurse cells consequently enlarge in cytoplasmic volume, the majority of which is transferred to the growing oocyte via the ring canals that connect to all 15 nurse cells, thereby facilitating growth of the oocyte during stages 11–12 of oogenesis [53–55].

Endoreplication as a mechanism to increase cellular volume is advantageous to cells that need to perform a secretory function, such as cuticle secretion of the ovarian follicle cells [56], or an absorptive function, as required by enterocytes that make up 90% of the cells of the adult *Drosophila* midgut [57,58]. Endoreplication has also evolved as a mechanism to rapidly respond to tissue damage in the adult epithelial barrier. Puncture wounds to the epidermis induce wound-induced polyploidization. The cells immediately adjacent to the wound undergo both fusion with neighbouring cells and endoreplication to produce large polyploid cells which function to seal the damaged epithelium [59–63]. This type of wound healing is not specific to the epidermis, as wounding of the intestinal epithelium induces EGFR-dependent endocycles to increase ploidy, and subsequent size, of enterocytes and thereby also heal a damaged epithelium [58].

## Polyploid proliferation

The induction of endoreplication is generally associated with an end point in a differentiation process as polyploid cells experience difficulties in attempting to re-enter the mitotic cycle. Multiple sets of chromosomes and centrosomes present a difficulty for accurate chromosome segregation leading to the process being error-prone and resulting in aneuploidy [40], which has also been observed to be facilitated by defects in maintaining chromosome alignment at the metaphase spindle due to a reduction of kinetochore-microtubule turnover in polyploid cells [64], and by extra chromosomes acting as a barrier to spindle pole coalescence [65]. Some cells are able to tolerate DNA replication errors by repressing proapoptotic gene expression [66], while ovarian follicle cells have evolved an ingenious mechanism to prevent the chromosomal instability that is associated with multiple centrosomes — they prevent centriole duplication during each S-phase of endoreplication and eventually eliminate them [67]. The endocycles that generate polyploid cells lack some of the checkpoints associated with cell cycle control resulting in frequent under-replication of heterochromatin [30,68]. All of these characteristics would seem to make mitosis of polyploid cells impossible during normal development, yet it has been observed to occur. Larval polyploid rectal cells avoid apoptosis during metamorphosis but proliferate to generate four rectal papillae of the adult rectum. The 8C larval cells undergo two error-prone mitotic divisions that commonly display lagging chromosomes and chromatin bridges resulting in aneuploidy [69]. This developmental strategy may only be possible due to the limited number of mitoses completed by larval rectal cells, allowing expansion of a tissue that functioned during larval life and has not developed from a specific imaginal-like set of cells specifically segregated prior to metamorphosis.

## Amitosis

**Figure 3.**
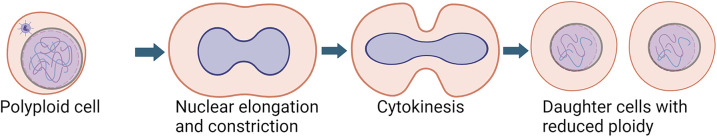
Amitosis — regeneration of diploid stem cells from polyploid enterocytes. Amitosis is a reductive division observed in polyploid cells that divide without generating a spindle or any of the features of mitosis. Nuclei elongate and constrict, along with a cytokinetic division that divides the cytoplasm and nucleus into two separate cells. When this occurs in the absence of DNA replication it results in progeny with reduced ploidy. Image was generated with Biorender.com.

A second, unusual form of polyploid proliferation, known as amitosis, has been observed to occur within enterocytes of the adult midgut following epithelial damage. Amitosis is a reductive cell division exclusive to polyploid cells, comprising of nuclear cleavage and cytoplasmic division without spindle formation, chromatin condensation or nuclear membrane dissolution (Figure 3), and results in daughter cells with approximately equal nuclear DNA content [70–72]. It is therefore distinct from mitotic division which produces daughter cells that are clones of the parent cell that underwent division. Amitosis has been best characterised in the ciliate, *Tetrahymena thermophila*[73,74]. Loss of chromosomes during amitotic division results in genetic heterogeneity and has been suggested as a mechanism to enhance genetic variation during vegetative growth of *Tetrahymena* [75]. Lucchetta and Ohlstein [76] determined that under conditions of starvation and regional loss of intestinal stem cells (ISCs) refeeding results in a rapid increase in ISC number yet an accompanying increase in ISC mitoses was not observed. Their study determined that diploid ISCs can be generated by amitosis of 4N differentiated enterocytes. Although this is a rapid mechanism to regenerate lost ISCs, unregulated chromosome segregation during amitosis (or ploidy reduction) can lead to the loss of heterozygosity and hence diploid ISCs homozygous for deleterious mutations could be formed. These errors, even if present at low frequencies, may have strong implications for tissue homeostasis and cancer initiation because such an inability to reproducibly pass on genomic information during amitosis will facilitate tumorigenesis. The findings hence elucidate a possibility that endocycling followed by amitosis can act as a cancer-initiating factor [72,76].

## Asymmetric division

**Figure 4.**
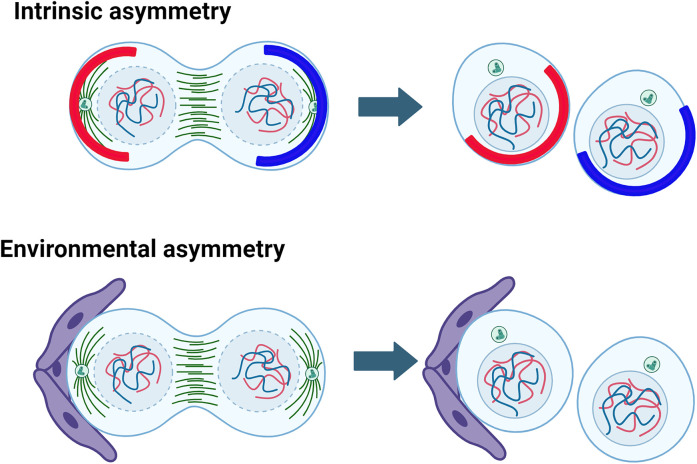
Asymmetric cell division — intrinsic asymmetry is observed in neural stem cells and environmental asymmetry in the testis germline stem cell niche. Stem cells may divide asymmetrically to produce daughters with different properties via an intrinsic asymmetry present in the parent cell such that different components are segregated to each daughter that influence cell fate. Asymmetric division may also be facilitated by the cellular environment such that niche cells serve to maintain a cell in a stem cell state, but a daughter cell leaves the influence of the niche is fated to differentiate. Image was generated with Biorender.com.

Adult stem cells that are harboured in specialized locations (niches) within tissues can maintain tissue homeostasis and regeneration by undergoing asymmetric cell division (ACD). Two daughter cells are generated from each ACD event, one will retain stemness that allows replenishment of differentiating cells while the other will initiate differentiation to support tissue function. The occurrence and the rate of ACD are both vital for maintaining tissue integrity and protecting from diseases and malignant transformation [77–80]. Depending whether it is governed by the niche, asymmetric stem cell division can be characterized as niche-dependent or niche-independent [79]. These modes of asymmetric division are also referred to as intrinsic asymmetry and environmental asymmetry (Figure 4).

## ACD of germline stem cells in *Drosophila* Gonads

In both *Drosophila* males and females, ACD in germline stem cells (GSCs) occurs shortly after primordial germ cells attain GSC identity through establishing cell-cell contact with somatic gonadal precursors [81–85]. Stemness is maintained either by receiving short-range signalling cues from the niche microenvironment or by autonomous regulation within GSCs [86–91].

At the tip of the *Drosophila* testis, a rosette of GSCs and somatic cyst stem cells (CySCs) adhere to a group of tightly packed somatic cells, the hub cells [92]. Both stem cell types undergo ACD to initiate the process of spermatogenesis. GSCs asymmetrically give rise to gonialblasts (GBs) that will be displaced from the hub and sequentially divide into 2, 4, 8, 16 spermatogonia and ultimately differentiate into spermatocytes [92,93]. The spermatogonial divisions are also characterised by incomplete cytokinesis such that they further develop as a group of 16 cells connected by ring canals and share a common cytoplasm. CySCs flank GSCs and together with hub cells form a microenvironment that supports GSC asymmetric division and self-renewal [86–90,94]. Somatic cyst cells that result from asymmetric division of CySCs encapsulate GBs and differentiating spermatogonia [95].

Regulation of spindle orientation that segregates chromosomes and various fate determinants into the two daughter cells is essential for asymmetric division [96]. In asymmetrically dividing cells orientation of the mitotic spindle determines if the cleavage plane is parallel to the niche so that one of the newly generated daughter cells will be displaced from the niche and initiate differentiation [96–99]. ACD of stem cells can be driven by epigenetic mechanisms at the centromere during spindle assembly [100] that can bias chromosome segregation. Intriguingly, during asymmetric division of GSCs, non-random segregation of sister chromatids has been observed [101] that could be associated with kinetochore and centromeric protein asymmetry, perhaps due to increased strength of attachment [102], within the daughter that retains stem cell identity [103]. Such a process would contribute to bias in microtubule attachment and segregation of sister chromatids carrying different epigenetic marks and may contribute to differential expression of differentiation and stemness genes [104–107].

GSC centrosomes are duplicated during S-phase, and the asymmetric behaviour of the original and duplicated centrosomes plays vital roles in cell adhesion between the hub and GSCs during ACD. After centrosome replication the daughter centrosome migrates towards the distal end of GSCs while the mother centrosome remains proximal to the hub to aid the cellular connection between the mitotic spindle and the hub-GSC interface through adhesion proteins and cytoskeleton proteins at the GSC cortex [98]. The colocalization of DE-cadherin at the hub-GSC interface serves as an anchorage for β-catenin and cortically localized adenomatous coli tumour suppressor homolog 2 (Apc2) that connects microtubules within the diving GSCs thereby enhancing adhesion of a region of GSC cortex to the hub, facilitating reception of niche signals from the hub cells [96]. Disruption of DE-cadherin and Apc2 function by mutation in spindle matrix protein, Megator (Mtor), can contribute to failure of centrosome mis-localization and spindle misorientation, resulting in failure of ACD and GSC loss [108]. Additionally, cell adhesion between GSCs and hub cells may generate polarity cues that intrinsically govern microtubule attachment and direct spindle orientation from the centrosome, and therefore asymmetric division [96].

Differentiating spermatogonia can resolve ring canals and dedifferentiate if GSCs are lost from the niche [109]. Live imaging of male GSC division has illustrated that producing differentiating daughter cells is hence not the inevitable and terminal outcome of asymmetric orientation of the division planes [110]. Sheng and Matunis [110] found that in the wildtype testis, GSC division oriented asymmetrically to the hub can result in both daughter cells having the same fates. This is accomplished either through ‘symmetric differentiation’, where GSC-GB pairs together dispatch from the hub and undergo differentiation, or ‘symmetric renewal’, where the initially displaced GB contacts back to the hub and obtains GSC identity [110]. Data suggest that ACD is more important for long-term function of GSCs than for maintaining GSC numbers, given that GSC identity is predominantly regulated by niche signals and GSC loss can be compensated by conversion of partially differentiated spermatogonia to GSCs [110,111]. Overall, this indicates that the GSC output is less restricted by how spindles are oriented but can be further altered depending on how germ cells adhere to the surrounding microenvironment.

ACD is also observed in female GSCs of the ovary. The adult *Drosophila* ovary is comprised of ∼16 ovarioles that can be considered as chains of differentiating germ cell cysts and associated somatic cells. Each ovariole regenerates from germline and somatic (or follicle) stem cells located within the most distal region of each ovariole, in a structure termed the germarium [112]. Two to three GSCs are found in the most apical region of the germarium where they contact somatic cap cells and anterior escort cells. These cells, in association with the more apically located terminal filament cells, serve to maintain the GSC population and regulate their differentiation [84]. This system was the first ever experimentally verified *in vivo* stem cell niche to be identified [113], some 20 years after the concept of the niche was theoretically proposed by Schofield [114]. As observed with the testis stem cell niche, multiple cell signalling pathways have been observed to tightly regulate maintenance and differentiation of GSCs [115–119]. We will here only discuss the role of bone morphogenic protein (BMP) signalling in regulation of GSCs as there are both similarities and significant differences in how this pathway is utilised in the female and male niches. The relative contributions of intrinsic and environmental cues to the regulation of ACD are somewhat difficult to evaluate but it appears that GSCs in males and females utilise both modes of regulation [100,120]. In addition to the aforementioned biased segregation of newly-replicated chromosomes [121], it is clear that in both sexes that BMP signalling from the niche plays an important role in preventing GSC differentiation [91,116,118]. Short-range BMP ligands secreted by niche cells repress the differentiation factor, Bag-of-marbles (Bam) in GSCs, resulting in the stem cell daughter that is displaced from the niche being fated for differentiation [122,123]. One might predict that loss of Bam would result in an accumulation of GSCs, and this is what is observed in *bam* mutant ovaries. However, the role of Bam is slightly different in the male germ line as its loss results in accumulation of cells connected by fusomes, suggesting that they enter a prolonged spermatogonial division period although the mutant germ cells also express some markers of GSCs [124,125]. Ectopic activation of BMP signalling outside of the niche can promote GSC self-renewal in the female germline but not in the male germline [126,127], indicating that although BMP activation is required for GSC maintenance in testes, it is not sufficient without the input from other factors. This example demonstrates that the ‘environmental asymmetry’ model of stem cell ACD may often result from integration of multiple factors and specificities of niche architecture.

## ACD of ISCs in the posterior midgut

It remains unclear whether an anatomical niche exists to regulate ACD in ISCs. In early developmental stages, peripheral cells (PCs) serve as a temporal niche for adult midgut progenitors (AMPs), maintaining them in an undifferentiated state. However, by the onset of metamorphosis, PCs degrade, leading AMPs to differentiate into ISCs [128]. Muscle sheath isolated from ISCs by a thin layer of basal membrane serves as a potential niche that is responsible for the homeostasis of ISC self-renewal and differentiation. The ligand Wingless (Wg) is exclusively expressed in the muscle sheath and autonomously activates the canonical Wg signaling pathway in ISCs and further controls the level of Notch activities to regulate ACD [129].

In the posterior midgut ISCs that undergo asymmetric division along the apical-basal axis primarily generate a basal ISC and an apical enteroblast (EB) or a basal precursor to an enteroendocrine cell (EE) and an apical daughter ISC. Guo and Ohlstein [130] suggest that Notch signaling regulates ISC ACD in a bidirectional manner the in basal ISC-EB and apical ISC-EMC doublets. Expression of Dl in basal ISCs and EMC induces high Notch signaling in EBs and low Notch signaling in apical ISCs, which facilitates EB-EC differentiation and restrains apical ISC differentiation respectively. A high level of Notch signaling is required for ISC commitment to differentiation [131] and loss of Notch signaling results in basal ISC and EE accumulation [130]. Such Notch-induced imbalances of ACD outcomes can promote tumorigenesis by interaction with other signaling pathways (reviewed by [80,132]).

## *Drosophila* neural stem cells as an example of niche-independent ACD

Neural stem cells, or neuroblasts (NBs), reside in larval brain hemispheres and execute asymmetric division along the apical-basal axis to gives rise to daughter cells destined for neuronal and glial differentiation [133]. Instead of being regulated by self-renewal cues from the closely attached niche, identity of daughter cells generated from NBs is intrinsically determined by asymmetric delivery of protein complexes to either the apical or basal cortex [78,134–141]. Such a process is tightly co-ordinated with centrosome migration and cytokinesis so that the dividing NBs cleaves at a specific cell plane to unevenly distribute cortical proteins [77,78,142]. *Drosophila* NBs have been utilized as a model to investigate intrinsic mechanisms that regulate ACD. Protein complexes such as the Par complex (aPKC-Par6-Baz) that segregate at the apical cortex are only inherited by daughter NBs and facilitate self-renewal [143]. Other proteins such as Numb, Miranda, Prospero and Lgl (lethal (2) giant larva) are sorted to the basal cortex and inherited by daughter cells that will undergo differentiation [143–145]. The translocation of Numb and Miranda to the basal cortex is controlled by phosphorylation mediated by aPKC [144], while the presence of Lgl at the basal cortex suppresses the activity of aPKC, ensuring that aPKC remains active only at the apical cortex [143]. Such a feedback loop between apical and basal complexes is required for regulation of localization of these proteins, which is vital for correct ACD. Either ubiquitous localization of aPKC at both apical and basal cortex [146] or cytokinesis at an incorrect plane that symmetrically cleave apical and basal protein complexes [147] is sufficient to induce failure in asymmetric division of NBs that contribute to overgrowth of NBs. Such a model serves as an explanation of the origin of larval brain tumours [78,80].

## Other unusual division mechanisms in the male reproductive system

Germ cells could be considered as the first known example of an unusual form of cell division as cells from both sexes undergo a reductive form of division termed meiosis to produce haploid gametes. Although male and female gametes are both produced via this process the regulation of meiosis differs between sexes [148]. Female germ cells undergo a standard meiosis whereby homologs pair via a synaptonemal complex and DNA crossovers (chiasmata) form to facilitate genetic exchange and alignment of chromosomes at metaphase I to ensure correct chromosome segregation [149–151]. Male spermatocytes do not develop a synaptonemal complex (although some components may be required for homolog pairing) and fail to form chiasmata or undergo recombination, but utilise a different mechanism, alternative homolog conjunction, to segregate chromosomes [152–154]. The timing of meiosis also differs between sexes as in males meiosis generates four haploid spermatids from each spermatocyte which mature into spermatozoa but female oocytes pause in prophase of meiosis I and subsequently arrest in metaphase I where they again arrest until signals from passage down the oviduct stimulate oocyte maturation and completion of meiosis [149].

Adult male *Drosophila* contain two testes that are connected via sperm storage organs, known as seminal vesicles, to a common ejaculatory duct [155]. Two accessory glands, analogous to the mammalian prostate, also connect to the duct and produce seminal fluid, including proteins that are essential for fertility [156,157]. Accessory glands contain two secretory cell types, main and secondary cells with distinct roles in fertility [158,159]. Both cell types are unusual in being binucleate and polyploid [160]. These cells develop during metamorphosis and the polyploidy is a result of endocycling. Accessory gland precursor cells undergo a nuclear division in the absence of cytokinesis to produce binucleate cells that then undergo an endocycle to produce cells that each contain two 4C nuclei [160]. Immediately prior to this non-cytokinetic division the penultimate mitosis produces adjacent cells connect by ring canals suggesting that there is a sequential truncation of cell cycles to result in binucleate cells [161]. An additional juvenile hormone dependent endocycle occurs after eclosion and is required for production of functional gland cells that retain the capacity to undergo further endocycles in response to hormonal signals [161].

**Figure 5.**
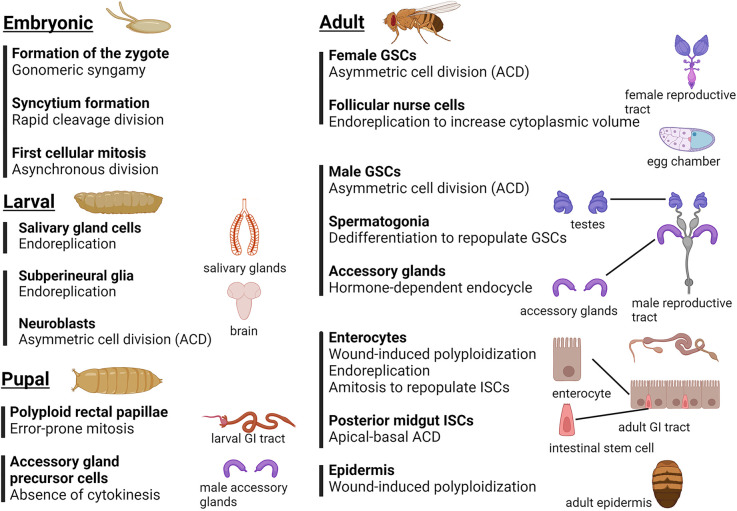
Overview — tissues that undergo variant cell cycles. *Drosophila* tissues at all stages of development exhibit variations in the standard cellular division mechanism. This review describes how haploid gametes combine via a gonomeric syngamy to produce the zygote, which then undergoes a series of rapid, synchronous nuclear cleavage divisions to form a syncytial embryo, which then cellularises and exhibits asynchronous cellular mitoses. Most larval tissues, such as the salivary glands and subperineural glia, undergo endoreplication to become polyploid, while regenerative tissues exhibit stem cell-like asymmetric cell divisions. Other variant division mechanisms in the pupal stage allow polyploid tissues to proliferate, and binuclear accessory gland cells in the male reproductive tract form due to suppression of cytokinesis after mitosis. Stem cells in both male and female adult reproductive tracts, and the midgut, exhibit asymmetric cell divisions (ACD), with spermatogonia capable of dedifferentiating to replace lost stem cells. These organs also contain specific cell types that utilise endoreplication to produce polyploid tissues. The midgut has evolved a unique mechanism to replace lost intestinal stem cells (ISCs) whereby polyploid enterocytes undergo a specialised reductive division, amitosis, to regenerate diploid ISCs. Polyploidization also facilitates wound healing within the adult midgut and epidermis. Image was generated with Biorender.com.

These examples demonstrate that a variety of modes of cell division are required to produce functional tissues in adult *Drosophila* (summarised in Figure 5), and that these tissues serve as excellent models for further investigation of cell cycle regulation.

## Perspectives

- Tissues of *Drosophila* exhibit a variety of DNA replication and cell division mechanisms that have the capacity to be experimentally analysed using the extraordinary genetic tools available in this organism. These studies will provide insight into the different modes of cell division that occur in all species. For example, polyploid giant cancer cells are present in over 30% of human tumours [162] where they can regenerate diploid cancer cells via amitotic mechanisms [163]. At present we have little knowledge of how this process is regulated.
- It has become apparent that differentiation of most tissues utilise non-standard mechanisms of cell division, particularly endoreplication to produce polyploid cells. The cells that continue to undergo mitosis in adult organs, e.g. gonadal and ISC populations often produce different daughters via asymmetric cell division so few cells in an adult *Drosophila* may undertake textbook cell cycles and division.
- Future work needs to be focused on how variant cell cycles are regulated, and if these modes are conserved across species. Analysis of the variety of cell and nuclear division mechanisms is likely to identify new methods to treat human disease. For example, polyploid giant cancer cells may be refractory to some standard chemotherapeutics as they do not divide mitotically but can utilise amitosis to re-seed diploid cancer cells. Thus, development of new drugs that target the amitotic process may be part of future combination therapies to suppress initiation of new tumour development.

## Open Access

Open access for this article was enabled by the participation of University of Melbourne in an all-inclusive *Read & Publish* agreement with Portland Press and the Biochemical Society under a transformative agreement with CAUL.

## Abbreviations

AMP: adult midgut progenitor
BAM: Bag-of-marbles
BBB: blood brain barrier
BMP: bone morphogenic protein
CySC: cyst stem cell
EB: enteroblast
GB: gonialblast
GSC: germline stem cell
ISC: intestinal stem cell
NB: neuroblast
PC: peripheral cell
SPG: subperineurial glia
